# How do quantitative studies involving people with dementia report experiences of standardised data collection? A narrative synthesis of NIHR published studies

**DOI:** 10.1186/s12874-024-02148-y

**Published:** 2024-02-16

**Authors:** Kate Gridley, Kate Baxter, Yvonne Birks

**Affiliations:** University of York, York, England

**Keywords:** Missing data, Data collection, Standardised measures, Dementia, Participant wellbeing

## Abstract

**Background:**

People with dementia are routinely included as research participants in trials and other quantitative studies in which they are invited to respond to standardised measures. This paper reviews the reporting of standardised data collection from people with dementia in reports published in the National Institute for Health and Care Research (NIHR) Journals Library. The aim was to understand how the administration of standardised, self-report measures with people with dementia is reported in NIHR monographs and what could be learnt from this about the feasibility and acceptability of data collection approaches for future studies.

**Methods:**

This was a systematic review with narrative synthesis. Broad search terms (Dementia OR Alzheimer*) were used to search the NIHR Journals Library website in December 2021. All studies that used (or intended to use) standardised measures to collect research data directly from people with dementia were eligible for inclusion. Information was extracted (where reported) on the process of data collection, dementia severity, levels of missing data and the experiences and reflections of those involved.

**Results:**

Searches returned 42 records, from which 17 reports were assessed as eligible for inclusion, containing 22 studies. Response rates from participants with dementia in these studies varied considerably and appeared to be related to dementia severity and place of residence. Little information was reported on the process of data collection or the reasons for missing data, and most studies did not report the experiences of participants or those administering the measures. However, there was an indication from two studies that standardised data collection could provoke emotional distress in some participants with dementia.

**Conclusions:**

Through this review we identified both variation in levels of missing data and gaps in reporting which make it difficult to ascertain the reasons for this variation. We also identified potential risks to the well-being of participants with dementia which may be associated with the content of standardised measures and the context of data collection. Open reporting of and reflection upon data collection processes and the experiences of people involved is essential to ensure both the success of future data collection and the wellbeing of study participants.

**Trial registration:**

Registered with Research on Research https://ror-hub.org/study/2905/.

**Supplementary Information:**

The online version contains supplementary material available at 10.1186/s12874-024-02148-y.

## Background

People living with dementia make up a significant proportion of the adult population using health and social care services [1] yet historically this group could be excluded from research participation [2]. Over the past two decades, a growing literature has both argued the importance of involving people with dementia as participants in research [3–6] and given practical advice about the best ways to achieve this [7–10]. However, the vast majority of good practice literature focuses on qualitative methods, emphasising the importance of flexibility and foregrounding the voice of the person with dementia [11–13], whilst relatively little has been written about the practice of involving people with dementia as participants in trials or other quantitative research [14, 15]. Despite this, standardised measures have been developed to collect quantitative data specifically from this group [16–20] and a number of existing measures have been validated for use with people with dementia [21]. Detailed monographs set out the development and psychometric properties of these measures, and some come with scripted instructions for their administration [18, 22], but very little has been published examining the process of data collection or the experiences of the people involved.

In the absence of an abundant literature on good practice in quantitative research with people with dementia, this paper reviews the reporting of data collection in published National Institute for Health and Care Research (NIHR) reports where standardised measures were used with people with dementia for research purposes. The review was conducted as part of a doctoral research project aiming to better understand the process and experience of structured data collection in a large study of people with dementia (the DETERMIND programme) [23]. The overall research explores what factors influence the answers given by people living with dementia to standardised measures, how these might change over time as dementia symptoms progress, and what the implications are for research incorporating standardised measures and the people involved. The aim of the review was to consider how the administration of standardised, self-report measures with people with dementia is reported in NIHR monographs and what can be learnt from this about the feasibility and acceptability of data collection approaches for future studies. A greater focus on acceptability in quantitative dementia research should be of interest to trial and other quantitative researchers, and to all those interested in the ethics of dementia research. Key debates in dementia trials research ethics have tended to focus on capacity, consent and use of proxy data [24] but there may also be ethical considerations related to the experience of research participation that are as yet unidentified.

### Standardised self-report measures for people with dementia

Questionnaires used in trials and cohort studies to measure outcomes, or assess health or psychosocial traits, are often standardised (with set wording, ordering of questions and answer scales) in order to ensure different scores reflect true differences between participants or time points rather than variation in the ways questions were asked [25]. When measures are administered face-to face, it is expected that interviewers will introduce and read each question to participants in the same way and instruct them to provide an answer in the required format in order to minimise the chances of interviewer bias [26, 27]. Since the early 2000s, research has indicated that people with dementia can (and should be enabled to) respond to such measures to appraise their own health and quality of life in this standardised way for research purposes [28–30]. A number of dementia specific measures have been developed; the most commonly used in published health research are DEMQOL [18] and QOL-AD [17]. These and other similar measures are referred to in this paper as ‘self-report’ to distinguish them from informant (family or professionals’) ratings of the person’s quality of life, proxy questionnaires (which typically ask family carers or professionals to consider how they think the person with dementia would score their own quality of life [31]) or observational measures such as the QuIS [32].

A number of reviews of the relative merits of different dementia specific and generic measures of quality of life have been published, but most tend to compare only the psychometric properties of measures, and although some do report rates of missing data and ‘feasibility’, there is rarely any mention of participant experience or focus on ability to respond to the items contained in the measures [33–36]. Whilst acceptability and respondent burden are important attributes of any measure [37] these tend not to be examined in the literature to the same degree as validity and reliability [38] even in dementia research where cognitive impairment and altered emotions may make this particularly relevant [12, 39]. Definitions of acceptability vary but tend to cover the degree to which participants find a measure difficult or distressing to complete, indicators for which can include refusal rates, response rates and administration time. As Fitzpatrick et al. note [38]:

*‘Pragmatically, trialists using patient-based outcome measures are concerned with the end result; whether they obtain as complete data from patients as possible… However, we need to consider the different components of acceptability in turn to identify sources of missing data.’* (p40)

Krestar et al. [40] did examine people with dementia’s ability to respond to different types of structured questions, concluding that participants with greater cognitive impairment struggled more when presented with bidirectional response categories (which contain two distinct concepts - such as ‘strongly disagree, disagree, agree, or strongly agree’) than when presented with scales which varied along only one dimension (such as “not at all, just a little, a fair amount, or a great deal”). Those who struggled were permitted to use simpler, dichotomous (yes/know) response categories, but most standardised measures do not allow this option. More recently, Cohen et al. [41] found a relationship between participants’ self-reported cognitive abilities and response times to standardised questions, with those with greater self-reported cognitive impairment taking longer to respond to questions with more syllables, those which contained abstract concepts, and those which required a degree of evaluation (as opposed to simple recall of frequency, for example). However, participants were recruited because they had one of five long-term neurological conditions, which may be accompanied by dementia, but acceptability for people with dementia in particular was not the primary focus of that study.

### The review

This paper presents a narrative synthesis of the reporting of standardised data collection from people with dementia in reports published in the English National Institute for Health and Care Research (NIHR) Journals Library [42]. The aim of the review was to explore the use of standardised, self-report measures with people with dementia as reported in the published monographs of research funded by the NIHR and available on the NIHR Journals Library website, focussing on the following indicators of experience, feasibility and acceptability:

- The level of missing data, in terms of both response rates to full measures and item completeness within individual measures, where this was reported
- The process of measure administration (how measures were used with people with dementia) and any reflections upon this process
- The views and experiences of the people involved, including:
  - ◦ Participants with dementia and their carers or other supporters
  - ◦ The research team, including the report authors and the researchers collecting data
- The impact of dementia severity on the experience of, and response rates for, standardised measures

## Methods

This paper presents a narrative synthesis of NIHR funded dementia research. A narrative synthesis is ‘*an approach to the systematic review and synthesis of findings from multiple studies that relies primarily on the use of words and text to summarise and explain the findings of the synthesis.*’ ([43], p5). We conducted systematic searches, selection, and data extraction to ensure comprehensive coverage (within tight boundaries) but approached the collation and presentation of findings narratively to allow for clarification and insight. The decision to focus on NIHR funded research reports was made for two reasons. Firstly, the NIHR is internationally renowned as a leader in public and patient involvement in research, so it would be reasonable to expect that studies funded by this body would exhibit good practice in data collection involving potentially vulnerable participants and those with additional communication needs. Secondly, a number of NIHR funding streams require the research to be published in detailed monographs adhering to strict guidelines which typically run to 50,000 words. These reports contain full details of study methods, as well as study findings and limitations and thus offer sufficient space to detail any observations or learning about the use of study measures and the experiences of people involved.

All dementia focussed research reports published on the NIHR Journals Library website [42] that reported the use of standardised self-report measures with people with dementia for research purposes were targeted for review. Here ‘self-report’ does not necessarily mean that participants responded to a question or measure independently (for example, online or on a paper questionnaire), indeed it is more common for older people and people with dementia to be asked to answer questions verbally in a structured face-to-face interview [26]. Thus, the term ‘self-report’ here means specifically that questions were expected to be answered directly by the person with dementia rather than by a proxy or informant, and scores were not based primarily on the ratings or judgement of another person.

### Search scope and dates

All final reports of studies listed in the NIHR Journals Library [42] involving standardised self-report data collection from people with dementia were in scope. Final searches were conducted on 17^th^ December 2021 with no restrictions on date of publication. The journals library was established in 1997, initially only covering the journal Health Technology Assessment, but by the date the searches were conducted the library comprised five NIHR open-access journals.

### Search terms and screening

Broad search terms (Dementia OR Alzheimer*) were selected in order to ensure that all potentially relevant reports were identified. No other search terms were used. Abstracts and (where the abstracts were not sufficiently clear) the full texts of all returned records were screened for eligibility.

### Inclusion criteria

- The study used (or intended to use) standardised self-report measures to collect research data from people with dementia

### Exclusion criteria

- No standardised self-report measures were used, or intended to be used, with people with dementia
- Study of carers only
- Measure development only
- Measures used for screening study population or routine clinical use only
- Review paper only

Where a report included multiple studies, one or more of which might meet the criteria, each individual study was screened for eligibility. Where a study included standardised data-collection from a subset of people with dementia, this was included, so long as at least some of the data were to be self-reported by participants with dementia themselves.

### Data extraction

Data were extracted from all included reports into an Excel spreadsheet under the following headings:

- Element of study involving standardised data collection from people with dementia
- Eligibility of participants with dementia
- Numbers of participants with dementia
- Severity and type of dementia
- Standardised outcomes measures to be completed by people with dementia
- Reporting of measure administration
- Data completeness and response rates
- Action to improve accessibility and acceptability for people with dementia
- Process evaluation/participants’ views on data collection
- Study teams’ comments/reflections on data collection with people with dementia

As this review formed part of a PhD study, the first author worked independently to select and review studies, with regular supervision by co-authors (YB and KB). After KG had completed data extraction, KB read and independently extracted data from two of the studies to cross check the data.

## Results

**Fig. 1:**
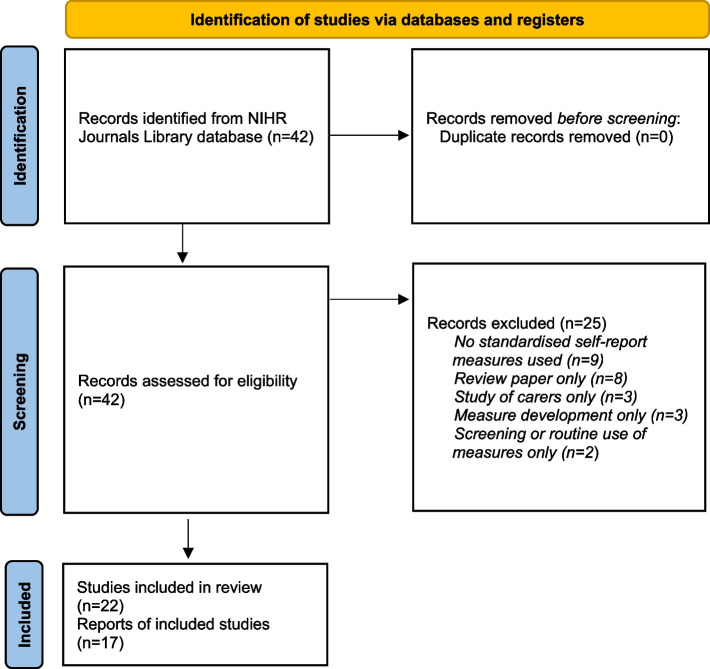
PRISMA flow diagram of assessment, exclusion and inclusion

A search of the NIHR Journals Library database using the terms Dementia OR Alzheimer* conducted on 17th December 2021 returned 42 reports out of a possible 2027. Figure 1 shows a PRISMA flow diagram with numbers of reports included and excluded, and reasons for exclusion. As some of the included reports contained more than one eligible study (for example, reports of programme grants), more studies were included (*n* = 22) than the total number of selected reports (*n* = 17).

Table 1 gives a full list of all self-report measures used with people with dementia in the 22 included studies and the primary outcome measure (where applicable). Some studies restricted participation to people with mild to moderate dementia, whereas others included people with all stages of dementia (including those with more severe symptoms). We found it useful to group studies that included participants with a similar level of dementia severity together, to enable response rates to be viewed in light of the mix of people involved. Table 1 groups studies under two headings:

- Studies collecting data from people with mild to moderate dementia only
- Studies collecting data from people with all stages of dementia

**Table 1:** Self-report measures used with people with dementia and response rates (studies arranged by dementia severity)

**Citation and included study**	**N with dementia, severity**	**Self-report measures, primary outcome**	**Response rates for self-report measures by time point**
**Studies collecting data from people with mild to moderate dementia only:**			
[44] Clare L, Kudlicka A, Oyebode JR, Jones RW, Bayer A, Leroi I, et al. Goal-oriented cognitive rehabilitation for early-stage alzheimer’s and related dementias: The GREAT RCT. Health Technol Assess. 2019 Mar 1;23(10):1–244 Included study: RCT of goal-oriented cognitive rehabilitation	*N* = 474 **Mild** to **moderate** Mean MMSE 23.82, ranging from 18 to 30	Self-reported measures: BGSI DEMQOL GSES HADS (depression and anxiety) RBMT TEA (with and without distraction) D-KEFS VF Primary outcome was self-reported goal attainment at 3 months	Baseline: (*N* = 474) BGSI: 474/474 (100%) DEMQOL: 472/474 (99.6%) GSES: 469/474 (98.9%) HADS D: 472/474 (99.6%) HADS A: 472/474 (99.6%) RBMT: 473/474 (99.8%) TEA: 463/474 (97.7%) TEA WD: 448/474 (94.5%) D-KEFS VF: 470/474 (99.2%) T1 (3 months): (*N* = 445) BGSI: 445/445 (100%) DEMQOL: 445/445 (100%) GSES: 439/445 (98.7%) HADS D: 444/445 (99.8%) HADS: A: 442/445 (99.3%) RBMT I: 444/445 (99.8%) RBMT D: 442/445 (99.3%) TEA: 429/445 (96.4%) TEA WD: 406/445 (91.2%) D-KEFS VF: 444/445 (99.8%) T2 (9 months): (*N* = 426) BGSI attainment 416 (97.7%) BGSI satisfaction 412/426 (96.7%) DEMQOL 417/426 (97.8%) GSES: 401/426 (94.1%) HADS D: 404/426 (94.8%) HADS A: 403/426 (94.6%) RBMT I: 411/426 (96.5%) RBMT D: 410/426 (96.2%) TEA: 397/426 (93.2%) TEA WD: 370/426 (86.9%) D-KEFS VF: 409/426 (96.0%)
[45] Clarkson P, Challis D, Hughes J, Roe B, Davies L, Russell I, et al. Components, impacts and costs of dementia home support: a research programme including the DESCANT RCT. Program Grants Appl Res. 2021;9(6):1–132 Included study: Pragmatic randomised trial of dementia home support	*N* = 468 **Mild to moderate** Participants were within one year of a memory clinic diagnosis with mild to moderate dementia (based on clinical assessment)	Self-reported measures: CASP-19 DEMQOL SMMSE EQ-5D-5L ICECAP-O Primary outcome measure was BADLS (not self-reported)	Baseline: (*N* = 468) CASP-19: 451/468 (96.4%) DEMQOL:446/468 (95.3%) SMMSE: 466/468 (99.6%) T1 (3 months): (*N* = 371) CASP 19: 358/371 (96.5%) DEMQOL: 350/371 (94.3%) SMMSE: 367/371 (98.9%) T2 (6 months): (*N* = 347) CASP-19: 322/347 (92.8%) DEMQOL: 323/347 (93.1%) SMMSE: 340/347 (98.0%) EQ-5D-5L and ICECAP-O (used for economic analysis) response rates not clearly reported (imputation used)
[46] Howard R, Zubko O, Gray R, Bradley R, Harper E, Kelly L, et al. Minocycline 200 mg or 400 mg versus placebo for mild Alzheimer’s disease: the MADE Phase II, three-arm RCT. Effic Mech Eval. 2020 Apr 24;7(2):1–62. Included study: RCT of Minocycline for mild Alzheimer’s Disease	*N* = 544 **Mild** Inclusion criteria specified SMMSE score of > 23 points. Mean SMMSE score at baseline was 26.4	Self-reported measures: SMMSE Primary outcome measure was BADLS (not self-reported)	Screening: (*N* = 544) SMMSE: 542/544 (99.6%) T1 (6 months): (*N* = 544) SMMSE: 498/544 (91.5%) T2 (12 ms): (*N* = 537) SMMSE: 453/537 (84.4%) T3 (18 ms): (*N* = 528) SMMSE: 420/528 (79.5%) T4 (24 ms): (*N* = 517) SMMSE: 403/517 (77.9%)
[47] Kehoe PG, Turner N, Howden B, Jarutyt L, Clegg SL, Malone IB, et al. Losartan to slow the progression of mild-to-moderate Alzheimer’s disease through angiotensin targeting: the RADAR RCT. Effic Mech Eval. 2021;8(19):1–72. Included study: RCT to study the effects of the antihypertensive drug losartan, in addition to normal care, compared with a placebo	*N* = 211 **Mild** to **moderate** Inclusion criteria specified that participants had an MMSE score of 15–28 at the consented eligibility assessment. Mean baseline MMSE score was 22	Self-reported measures: ADAS-Cog MMSE DEMQOL Primary outcome was difference in brain atrophy, measured using brain scans	Baseline: *N* = 211 ADAS-Cog: 207/211 (98.1%) MMSE: 209/211 (99.1%) DEMQOL: 211/211 (100%) 6 months: *N* = 204 ADAS-Cog: 194/204 (95.1%) DEMQOL: 202/204 (99.0%) 12 months: *N* = 197 ADAS-Cog: 182/197 (92.4%) MMSE: 192/197 (97.5%) DEMQOL: 186/197 (94.4%)
[48] Orgeta V, Leung P, Yates L, Kang S, Hoare Z, Henderson C, et al. Individual cognitive stimulation therapy for dementia: A clinical effectiveness and cost-effectiveness pragmatic, multicentre, randomised controlled trial. Health Technol Assess (Rockv). 2015;19(64):7–73 Included study: RCT of individual cognitive stimulation therapy for dementia	*N* = 356 **Mild** to **moderate** Clinical Dementia Rating: 70% CDR1 18% CDR 0.5 12% CDR 2 (One participant received a CDR score of 0) Mean MMSE 21.23	Self-reported measures: ADAS-Cog QOL-AD DEMQOL GDS-15 QCPR MMSE Primary outcome measures were ADAS-Cog and QOL-AD	Baseline: (*N* = 356) ADAS-Cog: 354/356 (99.4%) QOL-AD: 356/356 (100%) DEMQOL: 350/356 (98.3%) GDS-15: 350/356 (98.3%) QCPR: 348/356 (97.8%) MMSE: 356/356 (100%) Week 13: (*N* = 288) ADAS-Cog: 278/288 (96.5%) QOL-AD: 284/288 (98.6%) DEMQOL: 277/288 (96.2%) GDS-15: 276/288 (95.8%) QCPR: 281/288 (97.6%) MMSE: 285/288 (98.6%) Week 26: (*N* = 273) ADAS-Cog: 262/273 (96.0%) QOL-AD: 267/273 (97.8%) DEMQOL: 264/273 (96.7%) GDS-15: 262/273 (96.0%) QCPR: 269/273 (98.5%) MMSE: 268/273 (98.2%)
[49] Orrell M, Hoe J, Charlesworth G, Russell I, Challis D, Moniz-Cook E, et al. Support at Home: Interventions to Enhance Life in Dementia (SHIELD) – evidence, development and evaluation of complex interventions. Program Grants Appl Res. 2017;5(5):1–184 Included study: RCT of Maintenance Cognitive Stimulation Therapy (MCST)	*N* = 236 participants were randomised to the MCST or usual care group (half recruited from care homes and half recruited from community settings) **Mild to moderate** Inclusion criteria specified that participants had a CDR of between 0.5 to 2. ‘*Most of the sample had moderate dementia, with a mean MMSE score of 16.8 (SD 5.5) and a mean ADAS-Cog score of 34.3 (SD 12.9)*’ (pg30)	Self-reported measures: ADAS-Cog QOL-AD EQ-5D DEMQOL MMSE Primary outcome measures were ADAS-Cog QOL-AD	Response rate by measure not clearly reported. At follow-up 1 (3 months after baseline) 218 participants (92%) remained in the study and the reported response rate, excluding deaths, was 96% At follow-up 2 (6 months after baseline) 199 participants (84%) remained in the study and the reported response rate, excluding deaths, was 89%. Some details were given on how missing data were managed: ‘*Complete-case data analysis was used initially to establish the results, followed by the analysis with imputations.…. No data were imputed for those cases in which all assessments were missing. There were no participants missing for follow-up 1 who returned for follow-up 2*. *Primary analyses used an intention-to-treat basis, analysing participants according to the group to which they were randomised and using all data*’ (pp. 29–30)
[49] Orrell M, Hoe J, Charlesworth G, Russell I, Challis D, Moniz-Cook E, et al. Support at Home: Interventions to Enhance Life in Dementia (SHIELD) – evidence, development and evaluation of complex interventions. Program Grants Appl Res. 2017;5(5):1–184 Included study: Maintenance Cognitive Stimulation Therapy implementation study (observational)	*N* = 89 **Mild** to **moderate** Inclusion criteria specified that participants had a CDR of between 0.5 to 2.	Self-reported measures: QOL-AD MMSE Primary outcome measure was MMSE	Baseline: (*N* = 89) MMSE and QOL-AD scores for all 89 participants were reported. Follow-up 1: MMSE and QOL-AD scores reported for 62 participants (69.7%). Follow-up 2: MMSE scores available for 55 participants (61.8%), and QOL-AD scores available for 56 participants (62.9%).
[50] Woods R, Bruce E, Edwards R, Elvish R, Hoare Z, Hounsome B, et al. REMCARE: Reminiscence groups for people with dementia and their family caregivers - Effectiveness and costeffectiveness pragmatic multicentre randomised trial. Health Technol Assess. 2012;16(48):v–116. Included study: RCT of group reminiscence for people with dementia and carers	*N* = 487 **Mild** to **moderate** Inclusion criteria specified that participants were in mild to moderate stage of dementia based on CDR	Self-reported measures: QOL-AD EQ-5D QCPR One primary outcome measure was self-reported QOL-AD. The other was caregivers’ mental health	350 dyads ‘completed the study’. Response rates not clearly reported by time point.
**Studies collecting data from people with all stages of dementia:**			
[51] Allan LM, Wheatley A, Smith A, Flynn E, Homer T, Robalino S, et al. An intervention to improve outcomes of falls in dementia: The DIFRID mixed-methods feasibility study. Health Technol Assess. 2019;23(59):1–20 Included study: Feasibility study of a falls intervention	*N* = 11 **All stages eligible** Mean MoCA was 13.6, indicating moderate dementia (full breakdown of scores not reported)	Self-reported measures: EQ-5D-5L QOL-AD MFES MoCA Feasibility study, so no primary outcome	Baseline: (*N* = 11) EQ-5D-5L: 11/11 (100%) QOL-AD: 11/11 (100%) MFES: 11/11 (100%) MoCA:11/11 (100%) T1 (12 weeks) (*N* = 11) EQ-5D-5L: 10/11 (90.9%) QOL-AD: 10/11 (90.9%) MFES: 11/11 (100%) ‘*All self-reported and proxy EQ-5D-5L questionnaires that were completed had no missing data for any of the domains*.’ (p89)
[52] Banerjee S, Hellier J, Romeo R, Dewey M, Knapp M, Ballard C, et al. Study of the use of antidepressants for depression in dementia: The HTA-SADD trial- A multicentre, randomised, double-blind, placebo-controlled trial of the clinical effectiveness and cost-effectiveness of sertraline and mirtazapine. Health Technol Assess. 2013;17(7):1–43. Included study: Multicentre RCT of two antidepressants for people with dementia	*N* = 326 **All stages eligible** (one participant excluded because they had severe dementia SMMSE < 8) Mean (SD) SMMSE at baseline for the 3 groups: 18.2 (7.4); 18.5 (6.7), and 17.6 (6.0)	Self-reported measures: SMMSE EuroQOL VAS DEMQOL Primary outcome was depression measured by the CSDD (not self-reported)	Baseline SMMSE Placebo 82/111 (74%) Sertraline 79/107 (74%) Mirtazapine 90/108 (83%) Baseline EuroQOL VAS: Placebo 92/111 (83%) Sertraline 86/107 (80%) Mirtazapine 91/108 (84%) Baseline DEMQOL: Placebo 87/111 (78%) Sertraline 82/107 (77%) Mirtazapine 91/108 (84%) ‘Data availability’ reported separately for each group at baseline, but not clearly reported for T1 or T2.
[53] Bowen M, Edgar DF, Hancock B, Haque S, Shah R, Buchanan S, et al. The Prevalence of Visual Impairment in People with Dementia (the PrOVIDe study): a cross-sectional study of people aged 60–89 years with dementia and qualitative exploration of individual, carer and professional perspectives. Heal Serv Deliv Res. 2016;4(21):1–200. Included study: A cross sectional study of visual impairment in people with dementia	*N* = 708 **All stages eligible** Of those who were able to complete the SMMSE (*n* = 654): 21.1% (*n* = 138) had severe cognitive impairment 39.8% (*n* = 260) moderate 22.2% (*n* = 145) mild 12.7% (*n* = 83) very mild 4.3% (*n* = 28) no cognitive impairment	Self-reported measures: SMMSE Primary outcome was the result of eye examination	SMMSE 654/708 (92.4%) *‘Optometrists were able to perform an eye examination, although not necessarily a full eye examination, on all participants living in their own homes (group 1; n* = *389). Optometrists were unable to perform any part of the eye examination on eight participants living in care homes (group 2; n* = *319)*.’ (p40)
[45] Clarkson P, Challis D, Hughes J, Roe B, Davies L, Russell I, et al. Components, impacts and costs of dementia home support: a research programme including the DESCANT RCT. Program Grants Appl Res. 2021;9(6):1–132. Included study: Prospective observational study of dementia home support	*N* = 518 **Described as ‘later stage dementia’** Mean baseline SMMSE for each indicated moderate dementia on average: Basic care: 18.11 (SD 7.19) Int care: 15.74 (SD 6.87) Adv care: 16.20 (SD 6.29)	Self-reported measures: DEMQOL EQ-5D-5L SMMSE Primary outcome measure was BADLS (not self-reported)	Response rates not clearly reported. 389 people with dementia were interviewed at both baseline and 6 month follow-up, but it is not clear what proportion of each self-reported measure was responded to by these individuals, or how complete the measures were.
[54] Gathercole R, Bradley R, Harper E, Davies L, Pank L, Lam N, et al. Assistive technology and telecare to maintain independent living at home for people with dementia: The ATTILA RCT. Health Technol Assess. 2021;25(19):1–156 Included study: Pragmatic RCT of assistive technology and telecare	*N* = 495 **All stages eligible** Intervention group SMMSE at baseline (*n* = 248) 0–9: *n* = 23 (10%) 10–19: *n* = 79 (36%) 20–25: *n* = 87 (39%) 26–30: *n* = 32 (14%) Control group SMMSE at baseline (*n* = 247): 0–9: *n* = 34 (15%) 10–19: *n* = 96 (43%) 20–25: *n* = 74 (33%) 26–30: *n* = 19 (9%)	Self-reported measures: SMMSE EQ-5D-5L Primary outcomes were time to admission to care home and cost-effectiveness (using self-reported EQ-5D-5L)	Baseline: (*N* = 495) SMMSE = 444/495 (89.7%) T1 to T4: (*N* = 146) MMSE not reported EQ-5D-5L: reported in text as follows: ‘*Compared with the expected number of responses (given the number of assessments administered), approximately 10% of EQ-5D participant-reported index scores were missing at baseline. At 12 weeks, 13% of intervention participants’ and 20% of control participants’ responses were missing; at 24 weeks, 15% of intervention and 21% of control group participants’ responses were missing; at 52 weeks, 25% intervention and 31% of control group participants’ responses were missing. At 104 weeks, 22% of intervention and 34% of control group participants’ responses were missing*.’ (pg 40)
[55] Gridley K, Brooks J, Birks Y, Baxter K, Parker G. Improving care for people with dementia: development and initial feasibility study for evaluation of life story work in dementia care. Heal Serv Deliv Res. 2016;4(23):1–298. Included study: Feasibility study life story work with people with dementia (care homes)	*N* = 39 **All stages eligible** Dementia severity not assessed. Likely to be majority moderate to severe as most participants with dementia did not have capacity to give informed consent (43/59)	Self-reported measures: QOL-AD DEMQOL QCPR Feasibility study so no primary outcome	Baseline: (*N* = 39) QOL-AD: 25/39 (64%) DEMQOL: 12/39 (31%) QCPR: 13/39 (33%) Baseline *and* 1 months: QOL-AD: 23/39 (59%) DEMQOL: 12/39 (31%) QCPR: 7/39 (18%) Baseline *and* 2 months: QOL-AD: 23/39 (59%) DEMQOL: 12/39 (31%) QCPR: 5/39 (13%) Baseline *and* 6 months: QOL-AD: 18/39 (46%) DEMQOL: 12/39 (31%) QCPR: 4/39 (10%)
[55] Gridley K, Brooks J, Birks Y, Baxter K, Parker G. Improving care for people with dementia: development and initial feasibility study for evaluation of life story work in dementia care. Heal Serv Deliv Res. 2016;4(23):1–298. Included study: Feasibility study of life story work with people with dementia (mental health inpatient assessment units)	*N* = 12 **All stages eligible** Dementia severity not assessed. Likely to be severe as none had capacity to give informed consent	Self-reported measures: QOL-AD DEMQOL QCPR Feasibility so no primary outcome	Baseline: (*N* = 12) QOL-AD: 2/12 (16.7%) DEMQOL: 1/12 (8.3%) QCPR: 1/12 (8.3%) Baseline and 1–2 m: QOL-AD: 0/12 (0%) DEMQOL: 0/12 (0%) QCPR: 1/12 (8.3%) BASELINE AND 6 M: QOL-AD: 0/12 (0%) DEMQOL: 0/12 (0%) QCPR: 0/12 (0%)
[56] Iliffe S, Wilcock J, Drennan V, Goodman C, Griffin M, Knapp M, et al. Changing practice in dementia care in the community: developing and testing evidence-based interventions, from timely diagnosis to end of life (EVIDEM). Program Grants Appl Res. 2015 Apr;3(3):1–596. EVIDEM- C: mixed-method longitudinal study looking at incontinence	*N* = 34 **All stages eligible** Baseline MMSE scores (based on 14 completed measures) ranged from 2–26 (mean score 18)	Self-reported measures: MMSE DEMQOL No primary outcome measure	Baseline: (*N* = 34) MMSE: 14/34 (41.2%) DEMQOL: 7/34 (20.1%) T1, T2 and T3: No information. By the end year 3 only 2 participants with dementia remained: ‘*10 people with dementia died, six moved to care homes and one withdrew.*’ They were unable to follow-up 15 participants with dementia *‘due to time scale’* (p88)
[57] Kinderman P, Butchard S, Bruen AJ, Wall A, Goulden N, Hoare Z, et al. A randomised controlled trial to evaluate the impact of a human rights based approach to dementia care in inpatient ward and care home settings. Heal Serv Deliv Res. 2018 Mar;6(13):1–134. Included study: RCT of a human rights based approach to dementia care in inpatient wards and care homes	*N* = 439 (in total, but not all took part and not all from the start) **All stages eligible** Dementia severity unknown (only 13/332 completed ADAS-Cog at baseline). All participants were recruited from hospital wards and care homes	Self-reported measures: ADAS-Cog QOL-AD IDEA questionnaire Primary outcome measure was QOL-AD (self-report and proxy)	Not possible to calculate response rates from stated N of 439. Baseline: N for QOL-AD is reported as 265, made up of 102 self-reported scores and 163 proxy scores (these were analysed separately), but it is not clear how this relates to the overall sample of 439 people with dementia recruited to the study. IDEA questionnaire had 67 self-reported scores at baseline and 3 proxy scores. 260 ‘did not complete’ Between baseline and follow-up 122 new participants were recruited. T1(4 months): N for QOL-AD is reported as 287, made up of 93 self-reported scores and 194 proxy scores IDEA questionnaire had 36 self-report and 6 proxy scores. 391 ‘did not complete’
[58] Moniz-Cook E, Hart C, Woods B, Whitaker C, James I, Russell I, et al. Challenge Demcare: management of challenging behaviour in dementia at home and in care homes – development, evaluation and implementation of an online individualised intervention for care homes; and a cohort study of specialist community mental health car. Program Grants Appl Res. 2017;5(15):1–290 Included study: ResCare: Cluster randomised trial of online training for care home staff to deliver interventions for challenging behaviour in dementia. (Study 2)	*N* = 832 (555 with challenging behaviour) **All stages eligible** Nearly half had severe dementia at baseline: Baseline CRD: 0 = 4 (0.7%) 0.5 = 18 (3.2%) 1 = 88 (15.9%) 2 = 169 (30.5%) 3 = 273 (49.2%) Missing = 3 (0.5%)	Self-reported measures: EQ-5D QOL-AD Primary outcome measure was the NPI (not self-reported)	OUTCOMES STUDY (*N* = 832 at baseline, reducing to 658 at 4-month follow-up): Response rates were too low to justify imputation: ‘*Data for the residents’ responses were not imputed, as there were so many missing data (only 376, 174 and 214 out of the total 832 residents answered the EQ-5D index, VAS and QoL-AD, respectively).*’ (p57) ECONOMIC ANALYSIS (*N* = 428 at baseline) Calculations based on 206 self-reported EQ-5D scores (48.1%) and 165 self-reported QOL-AD scores (38.6%)
[58] Moniz-Cook E, Hart C, Woods B, Whitaker C, James I, Russell I, et al. Challenge Demcare: management of challenging behaviour in dementia at home and in care homes…. Program Grants Appl Res. 2017;5(15):1–290. Included study: FamCare: Observational cohort study of people with dementia and challenging behaviour living at home and their carers (Study 4)	*N* = 157 **All stages eligible** Over 90% had mild to moderate dementia at baseline: Baseline CDR: 0 = 5 (3.2%) 0.5 = 35 (22.3%) 1 = 59 (37.6%) 2 = 44 (28.0%) 3 = 14 (8.9%) T1 CDR: 0: = 3 (2.4%) 0.5 = 29 (23.0%) 1 = 45 (35.7%) 2 = 30 (23.8%) 3 = 19 (15.1%) T2 CDR: 0 = 6 (5.5%) 0.5 = 18 (16.5%) 1 = 39 (35.8%) 2 = 28 (25.7%) 3 = 18 (16.5%)	Self-reported measures: EQ-5D QOL-AD ICECAP-O QCPR Primary outcome was the Revised Memory and Behaviour Problems Checklist (not self-reported)	Baseline: *N* = 157 EQ-5D: 117/157 (74.5%) QOL-AD: 116/157 (73.9%) ICECAP-O: 115/157 (73.2%) QCPR: 115/157 (73.2%) T1 (mean 2.4 months from baseline): (*N* = 126) EQ-5D index: 87/126 (69%) EQ-5D VAS: 86/126 (68.3%) QOL-AD: 85/126 (67.5%) ICECAP-O: 86/126 (68.3%) QCPR: 86/126 (68.3%) T2 (mean 6.6 months from baseline): (*N* = 117) EQ-5D index: 74/117 (63.2%) EQ-5D VAS: 73/117 (62.4%) QOL-AD: 73/117 (62.4%) ICECAP-O: 72/117 (61.5%) QCPR: 72/117 (61.5%)
[59] O’Brien JT, Taylor J-P, Thomas A, Bamford C, Vale L, Hill S, et al. Improving the diagnosis and management of Lewy body dementia: the DIAMOND-Lewy research programme including pilot cluster RCT. Program Grants Appl Res. 2021;9(7):1–120 Included study: Pilot RCT of a management toolkit for Lewy Body Dementia	*N* = 131 **All stages eligible** The majority of participants had mild to moderate dementia at baseline based on reported MMSE data: Control group MMSE: Mean 20.1 Median 22 Interquartile range 17–25 Intervention group MMSE Mean 21.4 Median 22 Interquartile range 19–26	Self-reported measures: DEMQOL GDS MMSE MoCA EQ-5D-5L HADS Feasibility study, so no primary outcome	Baseline *and* 6 months: *N* = 109 DEMQOL: 86/109 (78.9%) GDS: 92/109 (84.4%) MMSE: 98/109 (89.9%) MoCA: 93/109 (85.3%) EQ-5D-5L: 89/109 (81.7%) HADS: 101/109 (92.7%)
[49] Orrell M, Hoe J, Charlesworth G, Russell I, Challis D, Moniz-Cook E, et al. Support at Home: Interventions to Enhance Life in Dementia (SHIELD) – evidence, development and evaluation of complex interventions. Program Grants Appl Res. 2017;5(5):1–184 Included study: RCT of a Carer Supporter Programme and reminiscence intervention	*N* = 289 dyads (people with dementia and carers). **All stages eligible** The majority had mild to moderate dementia at baseline (94% had CRD scores between 0.5 and 2, the remaining 6% had a CDR of 3).	Self-reported measures: MMSE QOL-AD EQ-5D DEMQOL HADS QCPR Primary outcomes measures were QOL-AD (self-reported and proxy) and carers’ health related quality of life	Response rates at follow up not clearly reported. Report sets out how missing data were handled in analysis but does not say how much there was at follow-ups, or from which measures: ‘*Multiple imputations at time points were conducted; however, no imputation was completed for a dyad if all measures were missing at a time point.*’ (p.82)
[60] Surr CA, Holloway I, Walwyn REA, Griffiths AW, Meads D, Kelley R, et al. Dementia care mapping™ to reduce agitation in care home residents with dementia: The epic cluster rct. Health Technol Assess. 2020;24(16):1–174 Included study: Cluster RCT of dementia care mapping in residential care settings (with a cross sectional element added at 16 months)	Initial sample *N* = 726 (all care home residents) **All stages eligible** 522/697 of the initial sample had moderately severe or severe assessments on the FAST scale at baseline N = 675 for the cross-sectional element at 16 months (414 from original cohort, 261 newly recruited). Of these, 570 had moderately severe or severe dementia based on FAST	Self-reported measures: QOL-AD (adapted for use with care home residents) EQ-5D-5L Primary outcome measure was CMAI (staff proxy rated)	Baseline: (*N* = 726) QOL-AD: 344/726 (47.4%) T1 (6 months): (*N* = 578) QOL-AD: 229/578 (39.6%) T2 (16 months): (*N* = 405) QOL-AD: 145/405 (35.8%) Cross section: (*N* = 666) QOL-AD: 269/666 (40.4%) EQ-5D-5L self-reported response rate not reported separately from proxy data.

Eight of the studies - in seven reports [44–50] - collected data from people with mild to moderate dementia (based on professional/carer assessment or scoring on a standardised self-report measure like SMMSE). The remaining fourteen studies – in 12 reports [45, 49, 51–60] - collected data from participants with all stages of dementia, including those with more severe symptoms. Some reports explicitly stated that dementia severity was assessed at baseline (and showed changes over time) whereas others reported severity as a static quality of the sample. A wide range of measurement tools was used across the included studies, most commonly to measure quality of life, cognition and various psychological characteristics. (These tend to be referred to by acronyms, so a glossary of measures is included as a supplementary file to aid comprehension.) Eight of the studies (in 7 reports: [44, 47–50, 54, 57]) had a self-report measure, to be completed by participants with dementia, as a primary outcome measure (usually alongside other self-report and carer or professional rated measures); seven studies (in 6 reports: [45, 46, 52, 54, 58, 60]) had a carer or professional rated measure as the primary outcome measure; two used ‘objective’ measures such as eye examinations or brain scans as the primary outcome [47, 53]; and five studies (in four reports: [51, 55, 56, 59]) did not identify a primary outcome. If response rates were not explicitly reported, we calculated these (where possible) from data provided in tables and accompanying text in the reports. Some reports amended follow-up sample sizes to reflect withdrawals, resulting in response rates appearing higher at follow-up than in studies employing an intention to treat approach. Sample size (N) at each time point has been included in Table 1 (if this information was clearly available from reports).

### Response rates, measure completeness and dementia progression

Table 1 illustrates that response rates (that is, the proportion of participants to complete each measure at each timepoint) varied considerably between studies, even where studies had similar designs. Studies with participants assessed as having mild to moderate dementia generally reported response rates of over 90% at baseline, but the degree to which this was maintained at follow-up varied (where reported). Response rates for studies that included people with more severe dementia varied more widely at baseline, from 20.1% (for DEMQOL) in a longitudinal study of a toolkit for incontinence [56], to 100% (for all baseline measures) in a feasibility study of a falls intervention [51]. Overall, studies which included people with all stages of dementia were less likely to report high response rates at any time point than studies which restricted participation to those with mild to moderate symptoms.

Response rates, or information from which a response rate could be calculated, were not always reported clearly by measure and time point [45, 49, 50, 52, 57, 60]. In an observational study of dementia home support for people with ‘later stage dementia’ [45], for example, the report states that 389 out of the 518 participants (75.1%) were interviewed at both baseline and 6 month follow-up, but it is not clear what proportion of each self-reported measure this group responded to at each time point. This is important as it does not necessarily follow that all 389 participants interviewed at both time points responded to all three of the self-report measures each time. We know from other studies that response rates to different measures can differ even within a time point. In a study of life story work with people with dementia in care homes, for example, 64% of participants responded to QOL-AD at baseline, but only 31% of those same participants responded to DEMQOL at baseline [55].

Response rates appeared to be associated with setting (i.e., whether participants were recruited from community or residential care or inpatient settings), but it is difficult to separate this from dementia severity, which in theory could be higher (reflecting the need for residential care) but in practice was not always measured. One study, for example, abandoned a measure (the IDEA questionnaire) after it transpired it was ‘*too cognitively complex*’ ([57], p35) for most participants with dementia to respond to. The dementia severity of participants in this study (all of whom were recruited from inpatient or residential care settings) is not known because only 13 participants out of a sample of 332 completed the cognitive test. Similarly, dementia severity was not formally assessed in two studies by Gridley et al. [55] which recruited from inpatient and residential care settings and had response rates ranging from 0% to 64%. Another study ([58], Study 2) which recruited people with dementia from residential care home settings had so much missing data that planned imputation was not conducted. People with all stages of dementia were included in this study, but nearly half (49.2%) were assessed as having severe dementia at baseline. Response rates were higher in the study by the same team which only recruited participants still living in the community. Whilst this latter study also included people with all stages of dementia, less than 9% of the community cohort had severe dementia at baseline.

Another large study which recruited exclusively from residential care settings [60] excluded all data collected directly from people with dementia from the analysis because of high levels of missing data, as the authors explained in their limitations section:

*‘Owing to the variability in the ability of care home residents with dementia to self-report on measures of BSC and QoL, the primary and secondary analyses were conducted using staff proxy-completed measures’* (p97).

By contrast, a study by Gathercole et al. [54] included people with all stages of dementia living in the community (not residential or inpatient settings), and reported higher response rates than the above studies, but lower than other studies which recruited from the community but restricted participation to people with mild to moderate dementia.

An RCT of individual cognitive stimulation therapy [48] reported very high response rates for multiple measures (typically close to 100%) which reduced only slightly over the 26-week follow-up period. In common with other large studies with high response rates, participants had mild to moderate dementia and were living in the community at baseline, and only people with capacity to consent and ‘*no major co-morbidities affecting participation*’ were eligible to take part. The authors note that tight eligibility criteria did restrict participation:

*‘In total, 1340 people were considered for recruitment to the study. From these, 356 were randomised and together constituted the final sample for the study. …Losses in 22% of cases were attributable to people with dementia not meeting the clinical criteria, indicating that this factor was, to some extent, a barrier to study recruitment.’* (p 5)

An implementation study of group based maintenance cognitive stimulation therapy (MCST) had similarly tight eligibility criteria [49], excluding people with severe dementia or any additional communication, physical or intellectual impairments, specifying that participants must ‘*have the ability to complete a cognitive and quality-of-life measure at three intervals over 1 year*’ (p. 51). This study applied intention to treat analysis, using all available information provided by participants with dementia at follow-up regardless of whether they completed the intervention programme, but it is not clear whether the reduction in available data over time (they reported QOL-AD for 89 participants at baseline, 62 participants at first follow-up and 56 participants at second follow-up) was the result of withdrawal from the study, other loss to follow-up or some participants declining (or finding it difficult) to respond to the measures. Response rates by measure for the other two eligible studies in this programme (an RCT of MCST and an RCT of a carer supporter programme and reminiscence intervention) were not clearly reported.

It was very rare for studies to report measure completeness, that is, what proportion of the items in individual measures were completed by participants. Allan et al. [51] did note that ‘*All self-reported and proxy EQ-5D-5L questionnaires that were completed had no missing data for any of the domains.*’ (p89) but this level of detail was very much the exception and perhaps only reported because theirs was a small feasibility study. Statements such as the following were more common, where authors set out how missing data were handled, without specifying how much there was or from which measures:

*‘Complete-case data analysis was used initially to establish the results, followed by the analysis with imputations. When individual data points were missing within a scale, data were imputed by using scale/subscale means according to the validated rules for the measures. When an outcome measure total score was missing, it was imputed using a multiple imputation regression model ...’* ([49], p29)

Without clear information about response rates by measure, measure completion, and the reasons behind missing data, it is difficult to compare approaches to data collection in different studies or ascertain possible explanations for problems encountered.

### The process of measure administration

Table 2 presents information reported from included studies on the process of measure administration: this included the views of participants on their experiences of taking part in the research (for example, from embedded process evaluations); and reflections by study teams on the data collection process or measures used. Ten studies (in nine reports) collected data from people with dementia face to face in participant’s own homes or another place convenient to them [44, 45, 47–51, 53, 58], six studies (in five reports) collected data from participants in care homes or hospital settings [55, 57–60], and the remaining six studies (in five reports) did not state where data were collected [46, 49, 52, 54, 56].

**Table 2:** Measure administration, participant views and author reflections

**Author (year) and study**	**Measure administration**	**Participants’ views on data collection**	**Author reflections on data collection processes and tools**
[51] Allan (2019) Feasibility study of a falls intervention	Data were collected during a home visit by one of three clinical trials assistants	Process evaluation involving people with dementia, carers and research workers looked at data collection processes. Research workers expressed concern about a) duration of assessments; b) wording of measures (use of double negatives in MFES, lack of clarity in QOL-AD)	The authors noted that, even though measures had good response rates, the wording was sometimes complex and difficult to explain to people with dementia. They stressed the importance of additional training for research workers to ensure a consistent approach and minimise missing data.
[52] Banerjee (2013) Multicentre RCT of two antidepressants for people with dementia	No information	Not reported	The authors comment that: ‘..*measurement error caused by the effect of cognitive impairment on domains such as memory, language and reasoning is a potential limitation. However, the study included only those measures best validated for use in dementia*.’ (p35)
[53] Bowen (2016) A cross sectional study of visual impairment in people with dementia	The study recruiter carried out SMMSE on the day that the participant was consented into the study Eye examinations were performed by an optometrist in participants’ homes and care homes	Not reported	The authors note that a considerable proportion of SMMSE assessments were not available ‘*owing to a range of factors, notably poor participant co-operation’* (p114) and highlight that unavailable SMMSE assessments were likely to come from patients with greater cognitive impairment.
[44] Clare (2019) RCT of goal-oriented cognitive rehabilitation	Assessments were completed by 15 trial researchers, all with backgrounds in psychology, nursing or clinical research. They received training in administering all outcome measures The researchers recorded assessment data manually during the participant (home) visits	As part of the process evaluation, an independent researcher interviewed participants in 3 sites about their experiences of the therapy sessions. The interviews did not appear to cover data collection processes directly, but data were collected during those therapy sessions. Overall, the therapy was received positively by both carers and people with dementia.	No specific reflections on the process of data collection. Table 57 lists all missing data in descending order of % missing. This includes ‘*participants who withdrew counted as missing data’* so combines overall study participation with response to particular measures. Percentages missing for self-report measures ranged from 0.4% at baseline for HADS and DEMQOL to 21.9% for the TEA distractor task at 9 months. The authors do not reflect on this in the full text.
[45] Clarkson (2021) Pragmatic randomised trial of dementia home support	Participants were interviewed face to face at home, often with carers present, by interviewers who had ‘*received online training about administering the standardised measures in a consistent and objective manner*.’ ([14]; p2735)	Embedded qualitative study [14] collected incidental comments from people with dementia about the experience of responding to standardised measures. Some participants felt anxious during the interview or were confused by questions and uncertain about how to reply.	The authors noted that the embedded study raised issues about the use of standardised measures that may be cognitively demanding for participants with dementia and said ‘*the research interview is not a neutral encounter*.’ (p29)
[45] Clarkson (2021) Prospective observational study of dementia home support	Participants were interviewed at home by research staff from participating trusts. Participants met their interviewer for the first time at this point.	Embedded qualitative study [61] focussed on carers’ incidental comments during data collection supplemented by a focus group of professionals (no-one with dementia).	No specific reflections relating to data collection from people with dementia (other than those noted above).
[54] Gathercole (2021) Pragmatic RCT of assistive technology and telecare	No information	There was an embedded ethnographic study, but this focussed on the use of assistive technology, not the research process or data collection.	The *‘burden of assessments’* is mentioned as one possible reason for some dyads not responding to measures despite continuing to participate in the trial, but there is no further explanation or discussion of this. The authors advise caution regarding generalisability as 8% of the sample at baseline (and 16–19% of the sample still included at follow-ups) did not participate in *any* interviews.
[55] Gridley (2016) Feasibility study of life story work with people with dementia (care homes)	Face to face data collection in care homes. Two researchers in the project team, supported by a research assistant, collected data at all time points.	Process evaluation involved qualitative interviews with 9 participants with dementia living in care homes, as well as staff and carers, about both the implementation of life story work and the acceptability of the research. Participants with dementia said they ‘didn’t mind’ answering questions, but some carers were concerned the person they cared for might have felt some anxiety when being questioned.	‘*Completion of outcome measures by people with dementia was challenging for a number of reasons, including: the capacity and frailty of the participants; the context in which data collection took place (care homes getting on with their daily routines)*’ (p69) ‘*The measures chosen were all designed to be completed by people with dementia but response rates leading to usable data were low and varied between the measures … The main reason for participants not completing a measure was that they were not able to understand and/or respond to the questions.*’ (p69)
[55] Gridley (2016) Feasibility study of life story work with people with dementia (mental health inpatient assessment units)	Face to face data collection in inpatient units. Two researchers in the project team collected data at all time points.	One participant with dementia residing in a mental health inpatient assessment unit was interviewed for the process evaluation (see above)	As above
[46] Howard (2020) RCT of Minocycline for mild Alzheimer’s Disease	No information	Not reported	Authors suggest that the low completion of SMMSE at follow-up was due to people on the higher dose withdrawing from the treatment and: ‘*Although the trial protocol specified that outcome assessments should be obtained irrespective of treatment compliance, this could not always be achieved despite the vigorous efforts of the trial team*.’ (p22)
[56] Iliffe (2015) EVIDEM- C: mixed-method longitudinal study looking at incontinence	No information	A feasibility study included interviews of carer participants about their experiences of data collection, but people with dementia were not interviewed.	There is no reflection on the small numbers of completed self-report measures in the discussion or limitations sections.
[47] Kehoe et al. (2021) RCT to study the effects of the antihypertensive drug losartan, in addition to normal care, compared with a placebo	Self-reported data were obtained during a face to face assessment by a researcher who, wherever possible, arranged to meet the participant where they felt most comfortable (e.g. at home or at the clinical research centre)	Embedded qualitative study looked at recruitment but not data collection	Authors note: Overall ‘*approximately 19% of the data or data sets were missing or incomplete, the majority of which related to the data collected from the various assessment tools used to collect some of the secondary outcomes’* Older participants were more like to have missing data
[57] Kinderman (2018) RCT to evaluate the impact of a human rights based approach to dementia care in inpatient ward and care home settings	Limited information, but authors note in discussion re QOLAD that ‘…*it quickly became obvious that the majority of people living with dementia in the care homes and wards visited were unable to complete the measure, even with assistance from skilled clinicians*.’ (p53)	Not reported	QOL-AD: Authors reflect that low QOL-AD response rates and heavy reliance on proxy measures (which consistently rated QoL lower than self-reports) call into question whether this was an appropriate measure to use in this context. IDEA Q: Authors note that, despite being developed collaboratively with people living with (the early stages of) dementia, staff and carers, the IDEA questionnaire was not an effective tool as it tended towards a floor effect, and was too complex for people with later stage dementia. ADAS-Cog: Most people refused this because of its length: ‘*On reflection, the use of a briefer screening assessment … might have yielded more useful results. Although these measures are less detailed than the ADAS-Cog, there is a greater chance that people would have engaged with them*…’ (pg59)
[58] Moniz-Cook (2017) Cluster randomised trial of online training for care home staff to deliver interventions for challenging behaviour in dementia (Study 2)	In most care homes, two researchers interviewed residents and care staff concurrently in separate rooms. In some instances, additional visits were arranged to complete interviews if, for example, participants became tired.	Not reported. Process evaluation included data from interviews with care home staff and a focus group with ‘stakeholders’ (not including anyone with dementia). The focus of both was the implementation of the intervention, not data collection processes.	A section of the report focusses on missing data but says little about the causes of missing data other than *‘The researchers endeavoured to collect as many data as they could. However, two types of missing data were inevitable: missing items within a measure and missing time points. Missing items were attributable to researcher error or participants declining to answer individual questions. When questionnaires had recommended rules for managing such missing items, these were applied*.’ (pg 40)
[58] Moniz-Cook (2017) Observational cohort study of people with dementia and challenging behaviour living at home and their carers (Study 4)	All interviews were conducted in the person’s home unless they requested an alternative location. Occasionally interviews were broken into chunks (either at the participant’s request or if the researcher deemed it appropriate)	Not reported. One person with dementia attended the stakeholder consultations (1/39 participants). The focus of discussion was the intervention and wider access to services. No mention of study methods or experiences of data collection.	As in study 2, the authors comment on the discrepancy between self-report and proxy QoL measures but do not reflect on the implications of this or low self-report response rates in the results, discussion, limitations or conclusion sections.
[59] O’Brien et al. (2021) Pilot RCT of a management toolkit for Lewy Body Dementia	The setting for the study was secondary care memory assessment and movement disorder services in England. All assessments were undertaken by members of the National Institute for Health Clinical Research Network	As part of an embedded qualitative study it is noted that ‘… *patients and carers highlighted some issues with question wording, typically with the same questions identified as problematic by clinicians’* (p39). No further information about the nature of the feedback or which questions were referred to.	In the limitations section the authors note: *‘There were occasionally some tensions between research paradigms, in particular in relation to managing qualitative feedback on question wording in the assessment toolkits, with the value given to ‘validated’ questions derived from clinical research*.’ (p44). No further explanation is given.
[48] Orgeta (2015) RCT of individual cognitive stimulation therapy for dementia	All research activities took place in participants’ homes. Showcards were used to support participants with dementia to respond to the measures. If participants felt uncomfortable with the assessment this was discontinued (and rescheduled where appropriate).	An embedded qualitative study explored experiences of 22 participants with dementia, but the focus was the intervention. No mention of participants’ experiences of data collection.	The authors reflect that the clinical criteria for inclusion of people with dementia were a barrier to recruitment.
[49] Orrell (2017) RCT of Maintenance Cognitive Stimulation Therapy (MCST)	No information (page 25 explains that: ‘*Half of the sample was recruited from care homes and half was recruited from community settings*’, but the report does not specify the context in which data collection took place).	Not reported	Commented that, in their analysis, ‘*DEMQOL seemed to be a more sensitive instrument than the QOL-AD for measuring change in quality of life in dementia*.’ Alternatively, ‘*the two measures may be measuring different aspects of quality of life*.’ (p37) The authors called for more research to explore the differences between these two measures further.
[49] Orrell (2017) Maintenance Cognitive Stimulation Therapy implementation study (observational)	Interviews with people with dementia were carried out by a researcher or staff member who was trained to undertake the assessment and had training in Good Clinical Practice and taking informed consent.	Three focus groups were conducted with 10 people with dementia and 5 staff members looking at the experience and effect of maintenance cognitive stimulation therapy. No mention of participants’ experiences of data collection.	No reflection on data collection, response rates or experiences of participants with dementia providing data.
[49] Orrell (2017) RCT of a Carer Supporter Programme and reminiscence intervention	Face to face interviews were held at ‘*times and venues organised to accommodate the carer’s needs and preferences*.’ (p78) The questionnaire for the person with dementia was always completed with the researcher.	Not reported. Participants are generally referred to as carers, although self-report data were collected from people with dementia.	No reflection on data collection, response rates or experiences of participants with dementia providing data.
[60] Surr (2020) Cluster RCT of dementia care mapping in residential care settings (with a cross sectional element added at 16 months)	The research took place in care homes. Little information is given about the data collection context other than to note that data were collected by ‘researcher interview’	Process evaluation focussed on implementation of the intervention, not data collection	Response rate was recognised as a problem and data collected directly from people with dementia was excluded from the analysis. This was noted as a limitation of the study: ‘*Owing to the variability in the ability of care home residents with dementia to self-report on measures of BSC and QoL, the primary and secondary analyses were conducted using staff proxy-completed measures’*
[50] Woods (2012) RCT of group reminiscence for people with dementia and carers	Face to face interviews were conducted in participants’ homes. Measures were arranged in a number of booklets. ‘*A second visit was sometimes made to complete assessments where an interviewee became tired, or where it was otherwise requested by participants or deemed appropriate by the assessor*.’ (p14)	No process evaluation, and this is identified as a limitation in the discussion	From the embedded study of EQ-5D the authors concluded: *‘Participants with dementia were able to complete the EQ-5D in a face-to-face interview, in line with evidence on suitability of this health-related quality-of-life instrument in this patient group.*’ (p49)

Overall very little information was given about the circumstances or activities that took place during data collection encounters. Typically, reports featured a statement such as ‘*outcomes were obtained during a face-to-face assessment by a researcher*….’ ([47], p15). Occasionally, a little more detail was offered, as in this example:

*‘The questionnaire measures were arranged into booklets, which facilitated their ease of delivery during the interviews. If a participant became tired, or if it was requested by participants or deemed appropriate by the researcher, an interview was occasionally broken off part-way through and then continued on another day.’* ([58], Study 4, p120)

Orgeta et al. and Woods et al. [48, 50] used very similar wording to explain that assessors occasionally arranged to return ‘*to complete assessments where an interviewee became tired, or where it was otherwise requested by participants or deemed appropriate by the assessor*’ ([50], p14). No further information was given in these reports about how often participants requested that an interview be paused and completed later, or why this might be ‘deemed appropriate’ by the researcher/assessor. The participants in these two latter studies had mild to moderate dementia. While a number of other studies used more measures and/or included participants with more severe dementia, they made no reference to breaking data collection sessions into more manageable chunks. It is unclear here whether such adjustments were not made, or just not reported.

Most studies did not report intervening to improve the accessibility or acceptability of data collection tools or processes for participants with dementia, other than to collect data face to face at a location acceptable to participants and employing trained research workers. Allan et al. [51] did reduce the number of questions in their health utilisation questionnaire and Orgeta et al. [48] used show cards (which typically present the answer scales visually) to support people with dementia to respond to the measures, with accompanying reports of high response rates. Surr et al. [60], on the other hand, used an adapted version of QOL-AD developed specifically for use in care homes which has ‘*simple language*’ and a four-response answer scale that is consistent across all questions, but still did not collect enough data directly from residents with dementia to enable their data to be used in the analysis. Similarly, Kinderman et al. [57] reported that people with dementia on hospital wards and in care homes received ‘*assistance from skilled clinicians*’ (p53) to answer QOL-AD, but most participants still did not complete this measure.

### The views of participants and reflections of study teams

Occasionally a report included a few lines about why participants did not attempt to complete assessments. Gathercole et al. [54] for instance note ‘*this could have been for several reasons, including disagreement with allocation, burden of assessments and delays in assessments being completed*.’ (p40) Unusually, the Bowen et al. [53] report sets out in some detail the reasons for missing scores for a specific measure (SMMSE) for 54 participants:

*‘These participants mainly comprised those for whom no coherent responses were obtained when attempting the test, and so could not be assessed using the SMMSE, and a small number who were unavailable, asleep or uncooperative on the day of recruitment, and so the test was not carried out.’* (p37)

Kinderman et al. [57] used ADAS-Cog in its standard form, but on reflection attributed the very low response rates achieved to the length of the measure, suggesting that in future more attention should be paid to the trade-off between the value of potential data from a measure, and the likelihood of obtaining enough data to be valuable:

*‘…the ADAS-Cog is often used in clinical trials because it can determine incremental improvements or declines in cognitive functioning. Despite this, it is a time-consuming assessment to complete (up to 45 minutes per person) and in reality the majority of participants refused to complete it.’* (p59)

Most of the reports did not include such candid reflections on the merits or otherwise of the measures selected for use. Neither did many include reflection on the data collection processes or the experiences of research workers or research participants. Process evaluations tended to focus on the process of implementing the studied intervention or recruiting participants, not data collection per se. However, five of the 17 reports did include some form of process evaluation or embedded study that touched on the process of data collection from people with dementia [45, 51, 55, 56, 59] EVIDEM-C [56] included interviews with carer participants about their experiences of data collection, but people with dementia were not interviewed. Allan et al. [51] interviewed participants with dementia, carers and research staff. The most common concern gleaned from their combined responses was that the baseline and follow-up assessments took too long to complete. O’Brien et al. [59] collected feedback from people with dementia as well as carers and clinicians on the measures to be included in their assessment toolkit and reported that *‘… patients and carers highlighted some issues with question wording*’ (p39). They noted ‘*tensions between research paradigms’* (p44), in particular the value ascribed to validated questions versus qualitative feedback from participants, but offered no further details.

With reference to field notes, Gridley et al. [55] identified a number of challenges inherent in collecting data from participants with dementia including the capacity and frailty of the participants; the context within which data collection took place (e.g. care homes where staff had other priorities); and the geographic location of the research settings, compared to that of the research team (given that data collection with people with dementia can be time consuming and require multiple visits). However, they also identified the closed-question format of the standardised measures as a key reason for low response rates. Clinical trial assistants (CTAs) interviewed for the Allen et al. [51] process evaluation reported concerns that the wording of some measures was difficult for participants with more advanced dementia to understand, for example because they contained double negatives. They also felt that some participants with dementia found the questions ambiguous and needed further explanation, which they had been trained not to give as this could impair the standardisation of the measure. The authors concluded that research workers like the CTAs require better training in the administration of standardised measures to ensure a consistent approach.

Clarkson et al. [45] had the most to say about the data collection context and process, and the influence of these on the data collected, producing an accompanying paper dedicated to reflecting on the research encounter. This paper was based on findings from an embedded qualitative study in which researchers audio-recorded the data collection process, revealing the dialogue surrounding the answers given to closed questions [14]. They noted that even people in the early stages of dementia ‘*struggled with the structured and standardised nature of the research interviews, finding them a linguistic and cognitive challenge*’ ([45], p15). They also noted the work that researchers had to undertake to determine whose perspective was being addressed, when family carers were present during data collection sessions with people with dementia.

### Emotional distress

The potential for standardised data collection to cause emotional distress in participants with dementia was explicitly identified as a risk in two of the reports [45, 55] and implied in a third [51]. While most of the reports made no mention of question content, Gridley et al. [55] noted the potential impact of sensitive or negative questions on participant wellbeing:

*‘…we found that, for example, asking people in quick succession whether they had lately felt sad (question 7), lonely (question 8) and then distressed (question 9) could trigger sadness. On one occasion (plus on two occasions in hospital wards) DEMQOL was abandoned specifically for this reason.’* ([60], p69)

Clarkson et al. [45] similarly noted that the measures they used addressed sensitive topics ‘*that could be distressing for people with dementia and their carers and difficult for interviewers to manage*.’ (p15). Their accompanying paper identified that some standardised questions could be ‘*very direct in probing potentially emotionally difficult aspects of life, particularly in the context of older age and deteriorating cognition*’ ([14], p2742).

Interviews with carers for the process evaluation by Gridley et al. also suggested that some people could find the experience of being questioned worrying in itself. Again, this concurs with the account of Abendstern et al. [14] suggesting that, for some, the structured interview as a whole appeared to cause anxiety:

*‘This was indicated in several ways including misunderstanding questions and showing uncertainty about how to reply, giving answers that they seemed to think the interviewer wanted, conveying feeling pressured to say the right thing, and forgetting things during the memory ‘test’…. Some participants expressed distress at the prospect of the interview itself, commenting that they were unsure about what to expect…’* (p2741)

The most common concern of those interviewed in the Allan et al. [51] process evaluation was the length of time it took to complete the baseline and follow-up assessments. However, through their illustration of this challenge it is evident that the data collection process in this study was also associated with, or may even have caused, emotional distress in some participants:

*‘…for the patients, it was a bit too much when you’re sat in the house. We only had, like, 90 minutes but I couldn’t do the first one in less than 2 hours because he kept getting upset and crying, it was very difficult.’*

*Professional 145, CTA (interview)*

([51], p93)

Such issues are generally not reported (or even recorded) in trials involving people with dementia. Together with the practice noted above of pausing data collection part way through (either if requested by participants or deemed appropriate by researchers) the issues highlighted by these three reports raise questions about participants’ experiences during data collection and the degree to which not only fatigue, but also emotional distress, may be features of the data collection process worthy of further investigation.

## Discussion

In this paper we presented a narrative synthesis of the reported use of standardised, self-report measures with people with dementia in 22 NIHR funded studies selected systematically from the NIHR Journals Library website. Response rates (where these could be ascertained) varied considerably and appeared to be related to dementia severity and place of residence, whilst measure completeness and patterns of item non-response were rarely reported. Overall, we found little reported information about the process of data collection from people with dementia (over and above basic setting and mode) or reasons for missing data. There was also very little information about the experiences of participants with dementia or those administering the measures. However, from the few instances where experiences were reported it seems that there may be risks to participants’ well-being associated with both the content of measures and context of data collection that are worthy of further consideration.

Despite some discrepancies in reporting, it was clear from the review that measures were not always completed in full at all time points and that some measures were not completed at all by some participants, even those still included in study samples. Such gaps are common in research; 100% response rates are rare [62] and missing data has been identified as a particular problem in research on ageing [63]. Some of the response rates reported in this review, however, seem to be considerably lower than would be expected for the general population, less than 50% in several cases, whilst in other cases they were close to 100%. Some of the apparent variation in response rates may be artefacts of reporting (for example, some studies amended sample sizes at follow-up in response to withdrawal, whilst others calculated response rates at follow-up using original sample sizes). However, it is clear that some studies faced real challenges in their attempts to obtain self-reported data from participants with dementia, which other studies appeared to avoid. The lack of detail on measure administration and participant experience means it was not always possible to determine the reasons underpinning these differences.

Studies that included people with more severe dementia tended to report more problems obtaining consistent response rates, supporting previous research where cognitive impairment has been shown to predict item nonresponse [15] or recourse to dichotomous (yes/no) answers [40]. Those which only included participants with milder cognitive impairment reported fewer problems and, where response rates were clearly reported, these were generally over 90% at baseline. In contrast, studies which included participants with all stages of dementia (i.e. including people with severe dementia) reported response rates ranging from 0 to 100%, with many under 75% or not reported. One approach used by some teams to minimise missing data was to apply tight eligibility criteria. However, while high response rates are desirable from a statistical perspective, restricting the eligibility criteria creates a trade off with generalisability, as the outcomes and perspectives of a group of people who could potentially be affected by the intervention under evaluation may not be included in the results [64, 65].

Little attention was paid in most reports to the potential risk to participants of emotional distress, despite previous flagging of this in the published literature, particularly in the context of qualitative research:

*‘All too often the person with dementia can be left with the feeling of not being able to do, not being able to remember or not reaching the right score, so they can feel excluded and a failure.’* ([66], p817)

Such risks have also been noted in quantitative data collection [26, 67–69] and there is some indication that standardised tests of cognitive impairment can be particularly problematic [21, 39, 70]. The bulk of literature highlighting the tension between the requirements of standardisation and the wellbeing of participants has focussed on data collection in clinical settings [71–73]. However, research participants may also experience feelings of anxiety and people with dementia may be particularly susceptible to emotional distress or agitation brought on or exacerbated by the research encounter [74–76].

Failing to understand the reasons behind missing data has implications for future successful data collection and appraisal of the appropriateness of measures. For example, if data are consistently missing for an item or measure because participants find it distressing to answer, the implications and possible remedies will differ from scenarios where items are skipped or measures dropped because they were found to be too cognitively challenging. The issues related to entire measures going unanswered by particular participants may also be different from the possible reasons behind individual missing items [62]. Patterns in item non-response may reflect problems with particular item wording specific to the communication and cognitive function of people with dementia which remain unaddressed without systematic examination [15]. Alternatively, missing data may be unrelated to the content or structure of the measures, but instead be the result of contextual factors such as care home practices: perhaps participants were not available at the allotted interview times [77], or carers were not available to provide support on the day. Certainly, in this review, studies attempting to collect data from participants with dementia residing in care homes or hospital wards appeared to achieve lower response rates than those collecting data from people residing in the community, perhaps reflecting known barriers to the undertaking of research in residential settings [78, 79].

A common solution proposed to the challenges of collecting research data directly from people with dementia is to use data from proxy measures alongside, or even instead of, self-reported data. However, in addition to the ethical issues of reliance on another person’s views in place of the person with dementia’s [69], some of the reports in this review flagged methodological issues inherent in this approach, such as the various relationships of proxies to participants [49], and the tendency for proxies to rate quality of life lower than people with dementia do themselves [57, 58]. This fits with the findings of multiple previous studies [30, 80, 81] and calls into question the ability of proxy measures to validly represent the views of people with dementia. At the very least, proxies may be reporting something conceptually different from the thing people with dementia themselves are reporting when asked about their ‘quality of life’ [82, 83].

An alternative solution would be to design methods or select measures more likely to be comprehensible, manageable and meaningful to people with dementia [84], that is, measures that are a better ‘fit’ for the people affected by the intervention so that they can answer for themselves [85]. Dementia specific quality of life measures like DEMQOL and QOL-AD were designed to do this, but the results of this review call into question the appropriateness of using even current dementia specific measures without additional support for some participants with dementia. Indeed, DEMQOL was only validated using data from people with mild to moderate dementia (data from people with an MMSE score of less than 10 were excluded from the analysis [18], and whilst it is commonly quoted that QOL-AD is suitable for use with people with an MMSE score as low as 3 (based on a 2003 study by Thorgrimsen et al. [86]), Kinderman et al. [57] struggled to use this measure with people with severe dementia in residential settings:

*‘Although it has been suggested that the QOL-AD can be usefully completed with some people with a MMSE score of as low as 3 (although it was originally suggested to be valid for use with people with MMSE scores of > 10), it quickly became obvious that the majority of people living with dementia in the care homes and wards visited were unable to complete the measure, even with assistance from skilled clinicians.’* (p53)

Without more detailed descriptions of what happens when researchers attempt to administer measures, and the individual items within those measures, it is hard to ascertain exactly which elements of measures, or research context, may be problematic and require attention.

Accounts from clinical settings have highlighted the, often marked, difference between the standardised conditions envisaged by those who design measures [87] and the realities of measure administration in practice. As Krohne explains:

*‘…test administrators must deal with interruptions, such as test-takers falling asleep, being in pain, not understanding the question, or consciously choosing not to respond to the question.’* ([88], p29).

Conventions in standardised interviewing [89, 90] along with the specific instructions for some measures (such as DEMQOL [18]) preclude the giving of support or explanations that are not in the script, even for participants with cognitive impairment. Yet there is evidence that standardisation in practice is difficult to achieve, even with participants with no cognitive impairment [91–94]. As Gobo, Giampietro, and Mauceri note [94] the standardised interview is ‘*an interaction that takes place in a social situation*’ (pXVII). Responses to accounts of researchers having difficulties adhering to standardisation tend to take the form of calls for better training (such as that in Allan et al. [51], but whilst training researchers to apply greater consistency in handling participants’ queries may reduce the chances of interviewer bias, this would not necessarily address participants’ anxieties, or any underlying problems with the measures themselves.

It is necessary to find a way through such dilemmas, as simply excluding specific groups from research participation because of their perceived vulnerability is no longer considered acceptable [95] and is certainly unacceptable to an increasingly politically conscious population of people living with dementia. Several key documents have been published in collaboration with people with dementia in recent years setting out, amongst other things, their right to be involved in research that concerns them [5, 6, 96]. Dementia care theorists advocate a personalised approach to working with people with dementia [97, 98] and a growing literature from the qualitative traditions have argued for greater flexibility in data collection, as Keady notes:

[Participants with dementia often have] *difficulties with linguistic, behavioural and cognitive functioning. Researchers therefore need to be creative and adapt their methods of data collection in order to address the individual needs of someone who is living with dementia.’* ([13], p2)

The principles of creativity and adaptability to the needs of the individual do not, however, sit well with the fundamentals of quantitative measurement, which rely on standardisation: essentially, inflexibility. This raises the question of whether quantitative data collection can feasibly be reconciled with the principles of best practice in dementia research. Evans et al. [99] looked at the relationship between reported quality of life scores and interviewer continuity and concluded that having a familiar person visit to collect follow-up data might influence results. The suggestion here is of a conflict between more person-centred approaches and data integrity, as opportunities to build rapport and put participants at ease might also lead to interviewer bias. However, those findings were based on exploratory secondary analysis of a completed study and the authors noted that ‘*characteristics, such as age, training, experience, warmth and ability to establish rapport, were not taken into account (given the lack of data)*’ ([99], p7). More attention must be paid to these factors, and the experiences of the people involved, to fully understand what influences scores and/or leads to missing data.

If compromises between standardisation and personalisation must be made, one solution proposed by Phillipson et al. [100] is to offer incremental levels of support including physical and emotional support, in addition to ‘easy read’ documentation, where this might facilitate the inclusion of people with a greater degree of impairment. Whilst this involves more flexibility than is usually permitted in standardised data collection, Phillipson et al. demonstrate that the provision of such tailored support enabled them to collect data from people who would not have been able to participate, or would have had large quantities of missing data, had they not been supported to respond, and their findings were richer because of this. However, this study only used one measure, the ASCOT, whereas some of the studies included in this review used multiple (up to 10 different) self-report measures with each person at each time point. It is likely that there would be a trade-off between the amount of tailoring of support practically achievable in a study and the number of measures used, with implications for the time and other resources required. It remains to be seen how applicable such an approach could be to a large trial or cohort study with multiple measures.

## Limitations

This review was conducted as part of a doctoral research project and as such is based primarily on the independent work of one researcher. However, two supervisors (both experienced senior health and social care researchers) were closely involved throughout and are co-authors on the paper. Moreover, the findings have been discussed more widely with colleagues working in the health and social care research field, including with those specialising in dementia research, with feedback integrated into our interpretation of findings. Nevertheless, it is recognised that working relatively independently, whilst necessary for doctoral studies, is a limitation in any review and the results should be read with this in mind.

The review only covered research published in a single, UK database and there may be learning from other types of research and research reporting not covered here. However, the NIHR is Britain’s largest funder of clinical, public health and social care research, and its Journals Library contains comprehensive, open access accounts of final, peer reviewed reports including methods and a full description of the results [101]. As such, the review offers a useful insight into the reporting of high status government funded dementia research and poses questions of relevance to the wider field.

## Conclusions

In this narrative synthesis we explored the use of standardised, self-report measures to collect data from people with dementia in NIHR funded dementia research and identified an important gap in reporting on the process of data collection and the experiences of participants. It seems that some studies, particularly those that recruited from residential care settings and/or included participants with more advanced dementia, were missing sizable quantities of data, but without clear reporting it is difficult to ascertain the full range of reasons for this or the specific links between dementia severity and responses to standardised measures. As noted by Hardy et al. [63], it is essential that authors are open in their reporting about the reasons for missing data so that we can both understand the implications of this and build upon learning to improve future research practice. In addition to potentially influencing the quality and quantity of data collected, learning from the few studies that did reflect openly about data collection processes indicated that the context and content of data collection could also influence the wellbeing of participants. It is imperative therefore that more attention be paid to the experiences of all those involved in quantitative data collection.

### Supplementary Information

**Additional file 1.** Glossary of Measures.

## Data Availability

The datasets analysed during the current study are available in the NIHR Journals Library https://www.journalslibrary.nihr.ac.uk/#/.
