# Supplementary material for: How do quantitative studies involving people with dementia report experiences of standardised data collection? A narrative synthesis of NIHR published studies
Source: BMC Med Res Methodol. 2024 Feb 16;24:43. doi: 10.1186/s12874-024-02148-y (PMC10870617; doi:10.1186/s12874-024-02148-y)
Supplement: Supplementary file 1 — Additional file 1. Glossary of Measures. [file 12874_2024_2148_MOESM1_ESM.docx]

**Glossary of Measures^[[1]](#footnote-1)^**

ADAS-Cog - Alzheimer’s Disease Assessment Scale – Cognitive: A standardised test to measure cognition. **Self-report.** (Richard C, David K, Ronald CP, Steven HF, Chris E, Michael G, Mary S, Linas B, David G, Chris C, Leon J. Development of Cognitive Instruments for Use in Clinical Trials of Antidementia Drugs. Alzheimer Disease & Associated Disorders. 1997;11:13-21.)

BADLS - The Bristol Activities of Daily Living Scale: A 20-item questionnaire designed to reveal the everyday ability of people who have memory difficulties. **Not self-report.** (Bucks RS, Ashworth DL, Wilcock GK, Siegfried K. Assessment of activities of daily living in dementia: development of the Bristol Activities of Daily Living Scale. Age and ageing. 1996 Mar 1;25(2):113-20.)

BGSI - Bangor Goal-Setting Interview: Offers a structured format for eliciting and measuring progress against individual goals. **Self-report.** (Clare L, Nelis SM, Kudlicka A. Bangor goal-setting interview manual. Exeter, United Kingdom: The University of Exeter. 2016 Dec.)

CASP-19 - Control, Autonomy, Self-Realization and Pleasure: A multidimensional measure of quality of life. **Self-report.** (Hyde M, Wiggins RD, Higgs P, Blane DB. A measure of quality of life in early old age: the theory, development and properties of a needs satisfaction model (CASP-19). Aging & mental health. 2003 May 1;7(3):186-94.)

CMAI - Cohen-Mansfield Agitation Inventory: A measure developed to assess agitation in older people with cognitive decline in nursing homes. **Not self-report.** (Cohen-Mansfield J. Instruction manual for the Cohen-Mansfield agitation inventory (CMAI). Research Institute of the Hebrew Home of Greater Washington. 1991;1991.)

CSDD - Cornell Scale for Depression in Dementia: Research worker rated assessment of depression in people with dementia. **Not self-report.** (Alexopoulos GS, Abrams RC, Young RC, Shamoian CA. Cornell scale for depression in dementia. Biological psychiatry. 1988 Feb 1;23(3):271-84.)

DEMQOL - DEMentia Quality Of Life: A patient reported outcome measure specifically designed to assess the health-related quality of life of people with dementia. **Self-report.** (Smith SC, Lamping DL, Banerjee S, Harwood R, Foley B, Smith P, et al. Measurement of health-related quality of life for people with dementia: development of a new instrument (DEMQOL) and an evaluation of current methodology. Health Technol Assess. 2005 Mar;9(10):1+)

D-KEFS (VF) - Delis–Kaplan Executive Function System (Verbal Fluency): A standardized set of tests to evaluate higher level cognitive functions in both children and adults. **Self-report**. (Delis, D. C., Kaplan, E., & Kramer, J. H. (2001). Delis–Kaplan Executive Function System. Odessa, FL: Psychological Assessment Resources.)

EQ-5D/EQ-5D-5L: Participants rate their health today against 5 dimensions (the original version has a 3 level scale, EQ-5D-5L has 5 levels) and a visual analogue scale (VAS). **Self-report**. (Herdman M, Gudex C, Lloyd A, Janssen MF, Kind P, Parkin D, Bonsel G, Badia X. Development and preliminary testing of the new five-level version of EQ-5D (EQ-5D-5L). Quality of life research. 2011 Dec;20:1727-36.)

EuroQOL VAS: An element of the EQ-5D that records self-rated health on a vertical visual analogue scale, where the endpoints are labelled 'The best health you can imagine' and 'The worst health you can imagine'. **Self-report.** (Herdman M, Gudex C, Lloyd A, Janssen MF, Kind P, Parkin D, Bonsel G, Badia X. Development and preliminary testing of the new five-level version of EQ-5D (EQ-5D-5L). Quality of life research. 2011 Dec;20:1727-36.)

FAST - Functional Assessment Staging Tool: Tool used to assess functional severity in people with dementia. **Not self-report**. (Reisberg B. Functional assessment staging (FAST). Psychopharmacology bulletin. 1988.)

GDS - Geriatric Depression Scale: A tool for detecting depression in older people. **Self-report.** (Yesavage JA, Brink TL, Rose TL, Lum O, Huang V, Adey M, Leirer VO. Development and validation of a geriatric depression screening scale: a preliminary report. Journal of psychiatric research. 1982 Jan 1;17(1):37-49.)

GSES - Generalized Self-Efficacy Scale: A 10-item psychometric scale designed to assess optimistic self-beliefs to cope with a variety of difficult demands in life. **Self-report**. (Schwarzer, R., & Jerusalem, M. Generalized Self-Efficacy scale. In J. Weinman, S. Wright, & M. Johnston, Measures in health psychology: A user’s portfolio. Causal and control beliefs (pp. 35-37). 1995. Windsor, UK: NFER-NELSON.)

HADS - Hospital Anxiety and Depression Scale: A scale designed to measure anxiety and depression in a general medical population. **Self-report.** (Zigmond AS, Snaith RP. The hospital anxiety and depression scale. Acta psychiatrica scandinavica. 1983 Jun;67(6):361-70.)

ICECAP-O: A capability-based measure of well-being on older people. **Self-report.** (Grewal I, Lewis J, Flynn T, Brown J, Bond J, Coast J. Developing attributes for a generic quality of life measure for older people: preferences or capabilities?. Social science & medicine. 2006 Apr 1;62(8):1891-901.)

IDEA questionnaire – identity, dignity, empowerment, autonomy: Human rights questionnaire developed with people with dementia for use in the Kinderman et al. (2018) NIHR study of a human rights based approach to dementia care in inpatient wards and care homes. **Self- report.** (Kinderman P, Butchard S, Bruen AJ, Wall A, Goulden N, Hoare Z, et al. A randomised controlled trial to evaluate the impact of a human rights based approach to dementia care in inpatient ward and care home settings. Heal Serv Deliv Res. 2018 Mar;6(13):1–134.)

MFES - Modified Falls Efficacy Scale: A 14-item tool designed to determine how confidently older people feel they are able to undertake activities on a scale of 0 – 10. **Self-report.** (Hill KD, Schwarz JA, Kalogeropoulos AJ, Gibson SJ. Fear of falling revisited. Archives of physical medicine and rehabilitation. 1996 Oct 1;77(10):1025-9.)

MMSE - Mini Mental State Examination: A widely-used measure of cognitive function. **Self-report**. (Folstein MF, Folstein SE, McHugh PR. “Mini-mental state”: a practical method for grading the cognitive state of patients for the clinician. Journal of psychiatric research. 1975 Nov 1;12(3):189-98.)

MoCA - Montreal Cognitive Assessment: A rapid screening instrument for mild cognitive impairment. **Self-report.** (Nasreddine ZS, Phillips NA, Bédirian V, Charbonneau S, Whitehead V, Collin I, Cummings JL, Chertkow H. Montreal cognitive assessment. The American Journal of Geriatric Psychiatry. 2003.)

NPI - Neuropsychiatric Inventory: Assesses 12 neuropsychiatric symptoms common in dementia. **Not self-report.** (Cummings JL, Mega M, Gray K, Rosenberg-Thompson S, Carusi DA, Gornbein J. The Neuropsychiatric Inventory: comprehensive assessment of psychopathology in dementia. Neurology. 1994 Dec 1;44(12):2308)

QCPR - Quality of Caregiver/Patient Relationship Scale: A 14-item scale measuring relationship quality, including level of warmth and level of criticism. **Self-report**. (Spruytte N, Van Audenhove C, Lammertyn F, Storms G. The quality of the caregiving relationship in informal care for older adults with dementia and chronic psychiatric patients. Psychology and Psychotherapy: Theory, Research and Practice. 2002 Sep;75(3):295-311.)

QOL-AD - Quality of Life in Alzheimer’s Disease scale: Dementia specific measure of quality of life. **Self-report.** (Logsdon RG, Gibbons LE, McCurry SM, Teri L. Assessing quality of life in older adults with cognitive impairment. Psychosomatic medicine. 2002 May 1;64(3):510-9.)

RBMT- The Rivermead Behavioural Memory Test: Tool designed to detect impairment of everyday memory function. **Self-report.** (Wilson B, Cockburn J, Baddeley A. TheRivermeadbehavioural memory test. Reading, UK: Thames Valley Text. 1985.)

SMMSE –Standardized Mini Mental State Examination: Standardised version of the MMSE, designed to impose strict guidelines for administration and scoring to improve reliability. **Self-report.** (Molloy DW, Standish TI. A guide to the standardized Mini-Mental State Examination. International psychogeriatrics. 1997 Dec;9(S1):87-94.)

TEA - Test of Everyday Attention: Tool for measuring selective and sustained attention and attentional switching**. Self-report.** (Robertson IH, Ward T, Ridgeway V, Nimmo-Smith I. The test of everyday attention (TEA). Bury St. Edmunds, UK: Thames Valley Test Company. 1994:197-221.)

1. Procedure to accompany Gridley, Baxter and Birks: *How do quantitative studies involving people with dementia report experiences of standardised data collection? A narrative synthesis of NIHR published studies* [↑](#footnote-ref-1)
